# Use of Bayesian Multivariate Meta-Analysis to Estimate the HAQ for Mapping Onto the EQ-5D Questionnaire in Rheumatoid Arthritis

**DOI:** 10.1016/j.jval.2013.11.005

**Published:** 2014-01

**Authors:** Sylwia Bujkiewicz, John R. Thompson, Alex J. Sutton, Nicola J. Cooper, Mark J. Harrison, Deborah P.M. Symmons, Keith R. Abrams

**Affiliations:** 1 Biostatistics Research Group, Department of Health Sciences, University of Leicester, Leicester, UK; 2 Genetic Epidemiology Group, Department of Health Sciences, University of Leicester, Leicester, UK; 3 Manchester Centre for Health Economics, Institute of Population Health, The University of Manchester, Manchester, UK; 4 NIHR Manchester Musculoskeletal Biomedical Research Unit, Arthritis Research UK Epidemiology Unit, School of Translational Medicine, University of Manchester, Manchester, UK

**Keywords:** Bayesian analysis, health technology assessment, meta-analysis, multiple end points, rheumatoid arthritis, surrogate end points

## Abstract

**Background:**

In health technology assessment, decisions about reimbursement for new health technologies are largely based on effectiveness estimates. Sometimes, however, the target effectiveness estimates are not readily available. This may be because many alternative instruments measuring these outcomes are being used (and not all always reported) or an extended follow-up time of clinical trials is needed to evaluate long-term end points, leading to the limited data on the target clinical outcome. In the areas of highest priority in health care, decisions are required to be made on a short time scale. Therefore, alternative clinical outcomes, including surrogate end points, are increasingly being considered for use in evidence synthesis as part of economic evaluation.

**Objective:**

To illustrate the potential effect of reduced uncertainty around the clinical outcome on the utility when estimating it from a multivariate meta-analysis.

**Methods:**

Bayesian multivariate meta-analysis has been used to synthesize data on correlated outcomes in rheumatoid arthritis and to incorporate external data in the model in the form of informative prior distributions. Estimates of Health Assessment Questionnaire were then mapped onto the health-related quality-of-life measure EuroQol five-dimensional questionnaire, and the effect was compared with mapping the Health Assessment Questionnaire obtained from the univariate approach.

**Results:**

The use of multivariate meta-analysis can lead to reduced uncertainty around the effectiveness parameter and ultimately uncertainty around the utility.

**Conclusions:**

By allowing all the relevant data to be incorporated in estimating clinical effectiveness outcomes, multivariate meta-analysis can improve the estimation of health utilities estimated through mapping methods. While reduced uncertainty may have an effect on decisions based on economic evaluation of new health technologies, the use of short-term surrogate end points can allow for early decisions. More research is needed to determine the circumstances under which uncertainty is reduced.

## Introduction

In health technology assessment (HTA), decisions are made about reimbursement of new health technologies. Such decisions are largely based on effectiveness outcomes, alongside cost and quality-of-life estimates. Effectiveness measures are usually estimated by meta-analyzing outcomes from randomized controlled trials (RCTs). The outcomes of interest (target outcomes), however, are sometimes not readily available. It may prove too costly to obtain the target clinical end point, or take an extended period of time to measure these outcomes, both of which conflict with the need for efficient and timely assessment of new technologies, especially in areas of health care that are considered a priority in society, such as cancer. Another reason for the target outcome not always being readily available is the wide diversity of alternative instruments measuring disease activity or response to treatment, as is the case, for example, in rheumatoid arthritis (RA), leading to a variation in the choice of an end point for a primary outcome in RCTs. Taking into account only the target outcome measure can lead to the amount of data included in the meta-analysis being radically reduced. In circumstances of absence of data on the target clinical outcome, surrogate end points are sometimes used in lieu of that outcome [1]. Alternatively, in some circumstances, data on the target end point may exist, but, in addition to this primary outcome, are also available either on alternative scales or measured by a surrogate outcome. Some manufacturers may choose a biomarker as a primary outcome, for example, when such an end point can be measured early, leading to a shorter time to licensing and hence quicker profit from the new pharmaceutical product. When a drug is conditionally licensed on the basis of a biomarker, data needed for HTA, such as overall survival in cancer treatments, are not available from the clinical trial evaluating this product. Multivariate approach to meta-analysis can allow us to include the data on the biomarker by combining it with data on the target outcome from other RCTs. Synthesizing the data from all sources of evidence can lead to a reduced bias [2] and potentially also the uncertainty [3].

Multivariate meta-analytic methods have already been developed to simultaneously synthesize evidence from correlated outcomes [4], [5]. In a recent review, Jackson et al. [6] describe advances in the development of the methodology of multivariate meta-analysis and discuss the advantages and disadvantages of the use of these methods. Bujkiewicz et al. [3] have recently developed a Bayesian multivariate meta-analysis (MVMA) framework for combining data from multiple correlated outcomes. One of the advantages of the multivariate approach that was exploited in this model was the incorporation of additional data by the inclusion of studies reporting multiple outcomes. In addition to this, the Bayesian aspect of this approach made it possible to incorporate external data in the MVMA model in the form of informative prior distributions, especially on correlations. This method, developed using an example in RA for combining data from studies reporting the Health Assessment Questionnaire (HAQ) and the Disease Activity Score 28 (DAS-28) measures, and the American College of Rheumatology (ACR) response criteria, allowed for extending the range of data that could be included in the analysis, thus reducing possible biases [2] and also led to a reduced uncertainty around the outcome of interest (the HAQ in this case). The aim of this work was to apply such a multivariate meta-analytic framework that allows evidence to be combined from studies that are a mixture of those reporting the target clinical outcome, an alternative clinical end point, or both (rather than reducing the number of studies to those reporting the clinical outcome of interest or including the surrogate end point alone in the economic model) to jointly estimate the effect of treatment on the HAQ. This is a flexible approach that permits the use of multiple outcomes and hence prevents valuable data from clinical trials from being discarded and potentially can lead to reduced uncertainty around the clinical outcome of interest.

Clinical effectiveness outcomes are often used to derive health-related quality-of-life or utility estimates, as part of the economic evaluation of the new health technologies. Increasingly, mapping methods are being developed to predict health state utility values from preference-based measures, such as EuroQoL five-dimensional (EQ-5D) questionnaire values, using disease-specific outcomes. For example, mapping methods have been applied to predict EQ-5D questionnaire values from 1) the St. George’s Respiratory Questionnaire in patients with chronic obstructive pulmonary disease [7], 2) functional assessment of cancer therapy-melanoma [8], 3) disease-specific quality-of-life scores in patients with migraine [9], 4) the Western Ontario and McMaster Universities Osteoarthritis Index in patients with knee osteoarthritis [10], and, finally, 5) using the HAQ in RA [11], [12].

In this article, we explore how using the multivariate Bayesian evidence synthesis can lead to a better estimation of the target clinical outcome in RA, in this case the HAQ. We then explore how reduced uncertainty around the HAQ, obtained from the MVMA, can ultimately lead to a better estimation of health utility such as the EQ-5D questionnaire obtained by a mapping technique.

## Methods

### Sources of Evidence

As our motivating example, we used a systematic review and meta-analysis that was carried out by Lloyd et al. [13] to investigate the effectiveness of the TNF-α inhibitors—etanercept, infliximab, and adalimumab—used sequentially (as a second-line treatment in patients who switched from one anti-TNF to another because of lack of response or adverse effects on the first) in the treatment of RA. The data from this systematic review are single arm (i.e., uncontrolled) because of the lack of comparative studies such as RCTs evaluating these treatments as second-line treatment. Outcome measures included in the analysis in this article were among the standard instruments used for measuring response to treatment in RA: changes from baseline in the HAQ (ΔHAQ) and DAS-28 (ΔDAS-28) measures, and 20% response to treatment measured by the ACR criteria (ACR-20). Data collected in this systematic review were used to investigate how the MVMA can be applied to simultaneously incorporate multiple outcomes of effectiveness in evidence synthesis. Table 1 gives details of the three outcomes that were reported in each of the studies within the systematic review. We will refer to these data as the “Lloyd data” throughout this article.

**Table 1:** Studies and outcomes included in the “Lloyd data.”

**Study**	**Mean (SE)**		**ACR-20*****r*****/n**
	**ΔHAQ**	**ΔDAS-28**	
Bennett (2005) [34]	−0.31 (0.13)	−1.7 (0.25)	–
Bingham (2009) [35]	−0.35 (0.05)	−1.6 (0.1)	85/188
Bombardieri (2007) [36]	−0.48 (0.02)	−1.9 (0.05)	486/810
Haraoui (2004) [37]	−0.45 (0.14)	–	14/22
Hyrich (2008) [38]	−0.12 (0.03)	–	–
Iannone (2007) [39]	0.15 (0.13)	–	–
Navarro-Sarabia (2009) [40]	−0.21 (0.07)	−1.1 (0.18)	–
Van der Bijl (2008) [41]	−0.21 (0.08)	−1.5 (0.25)	19/41
Buch (2007) [42]	–	−1.47 (0.18)	55/72
Cohen (2005) [43]	–	−1.87 (0.24)	–
Di Poi (2007) [44]	–	−2.1 (0.29)	–
Finckh (2007) [45]	–	−0.98 (0.18)	–
Hjardem (2007) [46]	–	−1 (0.11)	–
Laas (2008) InTol [47]	–	−1.17 (0.66)	–
Laas (2008) InEff [47]	–	−1.26 (0.35)	–
Nikas (2006) [48]	–	−2.4 (0.16)	18/24
Wick (2005) EA [49]	–	−1.9 (0.22)	7/9
Wick (2005) IA [49]	–	−1.3 (0.28)	19/27
Buch (2005) [50]	–	–	18/25
Karlsson (2008) [51]	–	–	172/337
van Vollenhoven (2003) [52]	–	–	12/18

ACR-20, 20% response to treatment measured by the American College of Rheumatology criteria; DAS-28, Disease Activity Score 28; EA, switchers from Etanercept to Adalimumab; HAQ, Health Assessment Questionnaire; IA, switchers from Infliximab to Adalimumab; InEff, switchers for reason of inefficacy; InTol, switchers for reason of intolerance; SE, standard error.

Additional sources of evidence were used to construct prior distributions for some of the parameters of the Bayesian evidence synthesis model. External individual patient data (IPD) were obtained from the British Rheumatoid Outcome Study Group trial, which was designed to assess the benefit of aggressive disease-modifying anti–rheumatic drug treatments in patients with established RA [14]. The trial, which recruited 466 patients with stable RA, assessed clinical outcomes (i.e., HAQ, DAS-28, and ACR-20) in two cohorts of patients managed by using either a regime focused on symptomatic control of pain and stiffness in the shared care setting or a more aggressive regime focusing on control of symptoms and joint inflammation in the hospital setting. (The British Rheumatoid Outcome Study Group trial found no difference between the aggressive and symptomatic treatment arms; therefore, this data set was used as a single cohort of 466 patients with established RA.) Another source of evidence, external summary data (ESD), was obtained from a systematic review of the anti–TNF-α inhibitors in biologically naive patients [15]. Both external sources of evidence (IPD and ESD) were used to construct prior distributions for correlations between outcomes as described below.

### Evidence Synthesis: Statistical Methods

In our motivating example, we aimed to model the summary data of the correlated outcomes from the Lloyd data by using an MVMA in a Bayesian form. We used a trivariate random-effects meta-analysis (TRMA), described in much more detail elsewhere [3], in the following form:

[1] $$\left(\begin{array}{l} \Delta {\mathrm{HAQ}}_{i}\\ \Delta {\mathrm{DAS}}_{i}\\ \mathrm{ACR}20_{i} \end{array}\right)\;\sim N\left(\left(\begin{array}{l} \mu_{Hi}\\ \mu_{Di}\\ \mu_{Ai} \end{array}\right),\left(\begin{array}{lll} \sigma_{Hi}^{2} & \sigma_{Hi}\;\sigma_{Di}\;\rho_{wi}^{HD} & \sigma_{Hi}\;\sigma_{Ai}\;\rho_{wi}^{HA}\\ \sigma_{Hi}\;\sigma_{Di}\;\rho_{wi}^{HD} & \sigma_{Di}^{2} & \sigma_{Di}\;\sigma_{Ai}\;\rho_{wi}^{DA}\\ \sigma_{Hi}\;\sigma_{Ai}\;\rho_{wi}^{HA} & \sigma_{Di}\;\sigma_{Ai}\;\rho_{wi}^{DA} & \sigma_{Ai}^{2}\; \end{array}\right)\right)$$

[2] $$\left(\begin{array}{l} \mu_{Hi}\\ \mu_{Di}\\ \mu_{Ai} \end{array}\right)\;\sim N\left(\left(\begin{array}{l} \beta_{H}\\ \beta_{D}\\ \beta_{A} \end{array}\right),\left(\begin{array}{lll} \tau_{H}^{2} & \tau_{H}\;\tau_{D}\;\rho_{b}^{HD} & \tau_{H}\;\tau_{A}\;\rho_{b}^{HA}\\ \tau_{H}\;\tau_{D}\;\rho_{b}^{HD} & \tau_{D}^{2} & \tau_{D}\;\tau_{A}\;\rho_{b}^{DA}\\ \tau_{H}\;\tau_{A}\;\rho_{b}^{HA} & \tau_{D}\;\tau_{A}\;\rho_{b}^{DA} & \tau_{A}^{2}\; \end{array}\right)\right)$$

which has a hierarchical structure: the correlated outcomes (ΔHAQ, ΔDAS, and ACR-20) in each study *i* follow a multivariate normal distribution and they are assumed to be the estimates of underlying correlated effects (*μ*_*Hi*_, *μ*_*Di*_, *μ*_*Ai*_) for the individual studies (the within-study models in Equation 1). These study-level effects are assumed to follow a common distribution (the between-study model in Equation 2). This meta-analytic model can be used for bivariate and univariate analyses by reducing the size of the matrices in the equations. The within-study variances ($\sigma_{Hi}^{2},\sigma_{Di}^{2},\sigma_{Ai}^{2}$) are assumed to be known (for studies reporting the relevant outcomes), but prior distributions need to be specified for the within-study correlations $\left(\rho_{wi}^{HD},\;\rho_{wi}^{HA},\;\rho_{wi}^{DA}\right)$ because none of the studies in the Lloyd data reported them. Prior distributions for the within-study correlations were constructed by using the external IPD described earlier. The elements of the covariance matrix of the between-study model—the between-study variances ($\tau_{H}^{2},\tau_{D}^{2},\tau_{A}^{2}$) and the between-study correlations ($\rho_{b}^{HD},\;\rho_{b}^{HA},\;\rho_{b}^{DA}$)—also need prior distributions to be placed on them. In our model, prior distributions on the between-study correlations were constructed by meta-analyzing ESD by using the trivariate model (see [1], [2]), but this time with noninformative prior distributions. Resulting posterior between-study correlations were then used as prior distributions to the between-study correlations in meta-analysis of the Lloyd data. To inform the between-study correlation by the ESD directly, the between-study model was parameterized in the form of the product of univariate conditional distributions (in contrast to using a multivariate Normal distribution, as in Equation 2, and a prior distribution on the precision matrix). Noninformative (half-normal) prior distributions are placed on the standard deviations (SDs) of these conditional distributions. ΔDAS-28 and ACR-20 are both conditional on ΔHAQ in this model. This parameterization assumes a conditional independence between ΔDAS-28 and ACR-20 that is equivalent to setting the partial correlation between ΔDAS-28 and ACR-20 to zero (but not the implied correlation; for details, see the Appendix in Supplemental Materials found at doi:10.1016/j.jval.2013.11.005). This assumption may sometimes be too strong, for example, in the circumstances in which the two variables are strongly correlated and then the implied correlation between them may be underestimated. This should not, however, have an effect on the estimation of the target outcome (ΔHAQ in this case). We have carried out a sensitivity analysis on the ESD to investigate the effect of the choice of the parameterization on estimates and in particular the resulting prior distributions for the between-study correlations, by using the spherical decomposition of the between-study covariance matrix [16], [17]. Details of this sensitivity analysis are included in the Appendix in Supplemental Materials found at doi:10.1016/j.jval.2013.11.005. The advantages of this approach along with all the details of the statistical modeling (including models in different number of outcomes) are reported elsewhere [3]. This evidence synthesis aimed to give an estimate of ΔHAQ from all available sources of evidence of the effectiveness (of studies reporting various outcomes), which can then be used to estimate the change from baseline in the EQ-5D questionnaire score, ΔEQ-5D.

### Mapping the HAQ Onto the EQ-5D Questionnaire

A number of methodological approaches to modeling utility estimates in RA for the purpose of economic evaluation have been developed. The estimate of the HAQ, a self-reported measure of physical function, is often used to predict EQ-5D questionnaire scores, an estimate of health-related quality of life, by mapping the HAQ score onto EQ-5D questionnaire scores [11], [12]. These methods often focus on the relationship between EQ-5D questionnaire scores and HAQ scores, usually expressed in a linear form [11], and have been compared with EQ-5D values based on patient responses in a number of data sets in RA [12]. In a systematic review, Lloyd et al. [13] found that very few studies reported the HAQ score, but there were other instruments used to assess patients’ response to treatment (ACR response criteria and the DAS-28 measure). The relevant instruments are summarized in Table 2. Using the multivariate evidence synthesis framework enabled us to take into account data from studies reporting those other measures of response to treatment in estimating the HAQ. The purpose of this article was to illustrate the potential effect of reduced uncertainty around the clinical outcome on the utility when estimating it from an MVMA. To do this, we modeled the relationship between the HAQ and the health-related quality-of-life measure, the EQ-5D questionnaire, in a linear form estimated by Chen et al. [15] from data reported in a study by Hurst et al. [18]: EQ-5D = *a* + *b* × HAQ, where the intercept $a\;\sim \;N(\mu_{a},s_{a}^{2})$ and slope $b\;\sim \;N(\mu_{b},s_{b}^{2})$, with means *μ*_*a*_ = 0.628 and *μ*_*b*_ = −0.327 and SDs *s*_*a*_ = 0.034 and *s*_*b*_ = 0.021, respectively (note that this approach assumed independence of intercept and slope). This relationship can be assumed to remain the same at any time point; hence, the relationship between the change from baseline in the EQ-5D questionnaire score and the change from baseline in the HAQ estimate can be modeled as ΔEQ-5D = *b* × ΔHAQ.

**Table 2:** Relevant instruments measuring disease activity and/or response to treatment in patients with RA.

**Measure**	**Description**	**Range (best to worst)**	**Form used in this article**
Disease Activity Score (DAS-28)	Composite of disease activity based on 28 swollen and tender joint count, ESR (or CRP), and patient global assessment [19]	0–9.3	Change from baseline (ΔDAS-28)
American College of Rheumatology (ACR) criteria	Indicated by % improvement in tender or swollen joint counts and three of the following five measures: acute-phase reactant (e.g., ESR/CRP), patient global assessment, physician global assessment, pain scale (e.g., pain VAS), disability/function (e.g., the HAQ) [20]	1, 0 (binary outcome)	20% improvement (ACR-20)
Health Assessment Questionnaire (HAQ)	Functional disability assessed on eight domains (dressing and grooming, rising, eating, walking, hygiene, reach, grip, activities) [21]	0–3	Change from baseline (ΔHAQ)

ACR-20, 20% response to treatment measured by the ACR criteria; CRP, C-reactive protein; ESR, erythrocyte sedimentation rate; RA, rheumatoid arthritis; VAS, visual analogue scale.

The change from baseline in the HAQ estimate, ΔHAQ, used in the above mapping equation, typically comes from a meta-analysis. As can be seen in Table 1, there are only eight studies reporting the HAQ in the Lloyd data, while there were many more studies investigating the effectiveness of the same treatment in the same type of patients but reporting DAS-28 and/or ACR-20. To be able to include all outcome data from all studies, we have used the multivariate approach to meta-analysis as described above.

### Software Implementation

All models were implemented in WinBUGS [22] in which the estimates were obtained by using Markov chain Monte Carlo simulation using 100,000 iterations (including 50,000 burn-in). All posterior estimates are presented as means, with measure of uncertainty for each estimate represented by the 95% highest probability density interval (HPDI) [23] obtained by using the BOA package [24].

## Results

We have applied the MVMA model to the Lloyd data to model simultaneously data on the HAQ, DAS-28, and ACR-20. To investigate the effect of including more data in the analysis on uncertainty around the HAQ estimate and consequently the EQ-5D questionnaire score, we explored results of the meta-analyses on three levels: using univariate random-effects meta-analysis (URMA) of ΔHAQ, bivariate random-effects meta-analysis (BRMA) combining ΔHAQ and ΔDAS-28, and finally TRMA by extending the data by including ACR-20. The prior correlations between DAS-28 and the HAQ obtained from the external data were as follows: $\rho_{wi}^{HD}$= 0.24 (95% HPDI 0.13–0.35) obtained from the IPD and $\rho_{b}^{HD}$ = 0.86 (95% HPDI 0.46–0.999) obtained from bivariate meta-analysis of the ESD. For the trivariate case, the prior within-study correlations, constructed by using the IPD, were $\rho_{wi}^{HD}$= 0.24 (95% HPDI 0.10–0.38), $\rho_{wi}^{HA}$= −0.13 (95% HPDI −0.29 to 0.0103), and $\rho_{wi}^{DA}$= −0.20 (95% HPDI −0.31 to −0.08), and the prior between-study correlations, constructed by using the ESD, were $\rho_{b}^{HD}$= 0.78 (95% HPDI 0.27–0.998) and $\rho_{b}^{HA}$= −0.14 (95% HPDI −0.80 to 0.56) (only the two correlations were required in the product normal parameterization of the between-study model).

**Fig 1:**
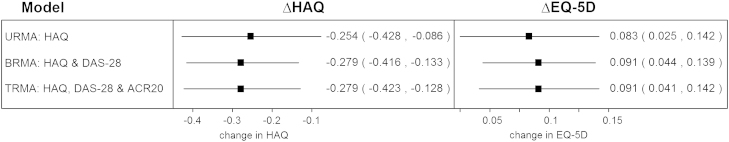
Estimates of change from baseline of HAQ, obtained from meta-analysis of HAQ only, HAQ and DAS-28 and finally HAQ, DAS-28 and ACR20, and resulting estimates of the change from baseline in EQ-5D.

Table 3 shows results of applying all the three models, including all univariate and bivariate models for the completeness of the results. Mapping estimate of ΔHAQ of −0.25 (95% HPDI −0.43 to −0.09), obtained from URMA of the eight studies reporting only HAQ outcomes, resulted in ΔEQ-5D equal to 0.08 (95% HPDI 0.025–0.141). Extending the evidence synthesis by 10 cohorts reporting ΔDAS-28 (but not ΔHAQ), by the use of BRMA, led to a shift in the estimate of the HAQ and the reduction in the uncertainty around the estimate to −0.28 (95% HPDI −0.41 to −0.14). This has also reduced the uncertainty around the estimate of ΔEQ-5D, which in this case was 0.09 (95% HPDI 0.041–0.138). This effect can be seen in Figure 1 in which horizontal lines represent 95% HPDIs. The intervals are reduced for the estimates of ΔHAQ and ΔEQ-5D when using BRMA compared with URMA. As can be seen in Table 3 and also in Figure 1, extending the evidence synthesis to TRMA by adding further three studies reporting ACR-20 did not contribute to a further reduction in the uncertainty around the estimates of interest (ΔHAQ and ΔEQ-5D). This is likely due to increased heterogeneity by introducing the three studies. More detailed description of results (related to the effectiveness outcomes only) are reported elsewhere [3]. Results of the bivariate meta-analyses of the HAQ with ACR-20 and of DAS-28 with ACR-20 are shown in Table 3 for completeness. Bivariate model of the HAQ and ACR-20 did not lead to reduced uncertainty around the HAQ similarly as for the trivariate case discussed above.

**Table 3:** Results of evidence synthesis from the three models: URMA, BRMA, and TRMA and resulting health-related quality-of-life estimates.

**Model**	**Number of cohorts/studies**	**ΔHAQ**	**ΔDAS-28**	**ACR-20**	**ΔEQ-5D**
HAQ only	8/8	−0.25 (−0.43, −0.09)	−	–	0.083 (0.025, 0.142)
DAS-28 only	15/13	–	−1.57 (−1.84, −1.31)	–	–
ACR-20 only	11/10	–	–	62% (53%, 71%)	–
HAQ and DAS-28	18/16	−0.28 (−0.41, −0.14)	−1.51 (−1.67, −1.35)	–	0.091 (0.044, 0.139)
HAQ and ACR-20	15/14	−0.31 (−0.5, −0.13)	–	60% (51%, 69%)	0.101 (0.039, 0.163)
DAS and ACR-20	19	–	−1.56 (−1.82, 1.30)	62% (52%, 71%)	–
HAQ, DAS-28, and ACR-20	21/19	−0.28 (−0.42, −0.13)	−1.51 (−1.70, −1.33)	61% (52%, 71%)	0.091 (0.041, 0.142)

ACR-20, 20% response to treatment measured by the American College of Rheumatology criteria; BRMA, bivariate random-effects meta-analysis; DAS-28, Disease Activity Score 28; EQ-5D, EuroQol five-dimensional; HAQ, Health Assessment Questionnaire; TRMA, trivariate random-effects meta-analysis; URMA, univariate random-effects meta-analysis.

## Discussion

We have applied a Bayesian meta-analytic framework to synthesize diverse sources of evidence and multiple end points in RA. Our aim was to increase the use of available data to estimate the effectiveness of second-line biologics used in the treatment of RA, measured by the change from baseline in the HAQ score and ultimately the health-related quality of life measured by the change from baseline in the EQ-5D questionnaire score. We used external IPD to construct prior distributions for within-study correlations and ESD for between-study correlations. We found that using this Bayesian approach to evidence synthesis of outcomes in RA, by combining the HAQ end point with DAS-28, leads to a 20% reduction in the uncertainty around the HAQ from −0.25 (95% HPDI −0.43 to −0.09) obtained from URMA of synthesizing data on the HAQ alone to −0.28 (95% HPDI −0.41 to −0.14) obtained from BRMA. The use of BRMA has also led to a reduced uncertainty, by 16%, around the health-related quality-of-life estimate, the EQ-5D questionnaire, from 0.083 (95% HPDI 0.025–0.141) when mapping the HAQ obtained from URMA to 0.091 (95% HPDI 0.044–0.139) when using the estimate of the HAQ from BRMA. Similar results have been obtained when using TRMA of the HAQ, DAS-28, and ACR-20. The inclusion of the third outcome, ACR-20, did not contribute to the HAQ estimate or further reduction of uncertainty around it. A 20% reduction in uncertainty around the effectiveness outcome may lead to a change in the cost-effectiveness outcomes, for example, alter the cost-effectiveness acceptability curve, leading to a change in a decision made on the basis of such analysis. Joint analysis of multiple outcomes may have advantages in many areas of decision making, for example, in risk-benefit analysis, in which multiple outcomes contribute to net benefit function and sometimes the risk function as well. For example, in a study on hormone replacement therapy by Minelli et al. [25], net benefit was constructed by adding the benefits measure by relative risk of hip fracture, menopausal symptoms, colorectal cancer, and endometrial cancer, whereas the overall harm was estimated by combining the relative risk of breast cancer, coronary heart disease, pulmonary embolism, and stroke. Each of these eight outcomes was estimated by a separate univariate meta-analysis. Modeling such outcomes jointly may lead to more precise estimates especially in circumstances in which outcomes are measured in RCTs that are powered with respect to one outcome but may be underpowered for others. This may also be the case when adverse events are included in the analysis while RCTs are not powered for such outcomes; therefore, there is a lot of uncertainty about them when including them in net benefit analysis.

When pooling data by means of the Bayesian random-effects meta-analysis, we allow for “borrowing of strength” across studies, which leads to “shrinkage” of the estimates from individual studies toward the overall mean (with reduced uncertainty around the estimates) [23]. In addition to this, an MVMA allows borrowing of strength across outcomes. This may lead to reduced uncertainty around the pooled estimates. It is difficult, however, to predict the extent of the gain in precision of the estimates. In fact, the inclusion of studies reporting additional outcomes through the multivariate approach can lead to higher heterogeneity in the data set (which is the case in our example in RA when extending the bivariate case by the third outcome), which can in fact lead to increased uncertainty. The issue of the potential gain in precision of the estimates obtained from multivariate models has been discussed previously. Nam et al. [4] observed that the gain is more likely to be noticeable when the data sets are small. Riley et al. [5] showed that the gain in precision is expected unless either the outcomes are not correlated or the data are complete on both outcomes, which are also reported with equal variance. In their recent review, Jackson et al. [6] reported that the gain in precision increases with increased within-study correlation, but noted that it can also depend on the between-study variance. While the factors driving the uncertainty around the estimates of the multivariate meta-analytic models seem complex and it is difficult to predict the gain in precision or whether its degree will be satisfactory to analysts, there are further advantages of using the multivariate scheme. Restricting the analysis to a single outcome may lead to ignoring data from clinical trials that either report treatment effect measured by a different instrument(s) or simply choose to report only positive and/or significant results. In the former case, the multivariate approach allows using more diverse sources of evidence and hence prevents relevant data from being discarded. In the latter case, in the presence of outcome reporting bias, the multivariate approach can lead to a more appropriate estimate of the clinical outcome as has been shown by Kirkham et al. [2]. This may apply to the case of RA in which, due to the availability of a number of instruments for measuring the disease activity or response to treatment, authors may choose to report only those outcomes whose estimates are positive and/or significant. The pooling of multiple outcomes through the multivariate approach can obviously be achieved by means of frequentist analysis. The Bayesian approach, however, brings additional advantages of incorporating external evidence in the form of prior distributions. Individual patient data (used in our example to construct prior distributions for within-study correlations) are not always available. Other sources of evidence, however, can be used to inform the parameters of the model. The Bayesian approach, for example, allows elicitation techniques to be used to take into account experts’ opinions in the form of prior distributions in a model. For example, methods for inclusion of expert opinion on bias in the synthesis of studies were developed by Turner et al. [26]. Implementing such Bayesian analysis requires the use of specialist software such as WinBUGS, which can be viewed as a limitation by analysts. MVMA can also be conducted, for example, in Stata by using the mvmeta command [27] or in R mvmeta package [28] in a frequentist approach. WinBUGS, however, provides a suitable software environment for implementing a full Bayesian framework with the use of external evidence and can be interfaced with R, which is convenient when decision models are developed in R [29].

In this multivariate approach to evidence synthesis, studies reporting various relevant measures associated with an outcome of interest can be included. Outcomes can include surrogate end points, so far mostly used instead of the primary outcome of interest when there is no available evidence on the target outcome [1]. In the method described here, the surrogate outcomes are used alongside the primary outcome, and therefore the analysis is not restricted to a single primary outcome or a surrogate end point. The main interest of this research is in uncertainty (and magnitude of the effect) in terms of the HAQ and also the EQ-5D questionnaire because it is this outcome that is used for decision making. In our example, none of the studies in the Lloyd data reported the EQ-5D questionnaire and hence the linear mapping was chosen to estimate the EQ-5D questionnaire from the HAQ. When some of the studies included in evidence synthesis report the EQ-5D questionnaire, however, the MVMA can include the EQ-5D questionnaire as one of the multiple outcomes and the pooled effect is then estimated simultaneously with the clinical outcomes (borrowing strength from data across studies and outcomes). Clinical outcomes can then be considered surrogate end points to utility. This approach may be preferable to linear mapping because regression models are often developed on the basis of a small amount of data. Cooper et al. [30] have shown that the data quality (together with quantity, and therefore uncertainty) can be least for utility estimates (e.g., the EQ-5D questionnaire), especially compared with effectiveness, and, therefore, in the absence of better direct utility data, the methods considered here may at least reduce uncertainty in the decision problem.

In our view, the approach described here is an important step toward the inclusion of surrogate end points in HTA, especially in the early stages of the drug development process when the target outcome is not readily available. Modeling simultaneously data on surrogate (potentially short-term outcome) and the ultimate end point can increase the available evidence, allowing analysts and decision makers an early assessment of new technologies. Surrogate end points play an increasingly important role in drug development as reported by the European Medicines Agency [31] and HTA, and their use will need to increase in HTA as reimbursement decisions are taken closer to regulatory ones [32] and RCTs become shorter in duration [33].

## References

[1] Taylor R.S., Elston J. (2009). The use of surrogate outcomes in model-based cost-effectiveness analyses: a survey of UK Health Technology Assessment reports. Health Technol Assess.

[2] Kirkham J.J., Riley R.D., Williamson P.R. (2012). A multivariate meta-analysis approach for reducing the impact of outcome reporting bias in systematic reviews. Stat Med.

[3] Bujkiewicz S., Thompson J.R., Sutton A.J. (2013). Multivariate meta-analysis of mixed outcomes: a Bayesian approach. Stat Med.

[4] Nam I.-S., Mengersen K., Garthwaite P. (2003). Multivariate meta-analysis. Stat Med.

[5] Riley R.D., Abrams K.R., Lambert P.C. (2007). An evaluation of bivariate random-effects meta-analysis for the joint synthesis of two correlated outcomes. Stat Med.

[6] Jackson D., Riley R., White I.R. (2012). Multivariate meta-analysis: potential and promise. Stat Med.

[7] Starkie H.J., Briggs A.H., Chambers M.G., Jones P. (2011). Predicting EQ-5D values using the SGRQ. Value Health.

[8] Askew R.L., Swartz R.J., Xing Y. (2011). Mapping FACT-melanoma quality-of-life scores to EQ-5D health utility weights. Value Health.

[9] Gillard P.J., Devine B., Varon S.F. (2012). Mapping from disease-specific measures to health-state utility values in individuals with migraine. Value Health.

[10] Xie F., Pullenayegum E.M., Li S.C. (2010). Use of a disease-specific instrument in economic evaluations: mapping WOMAC onto the EQ-5D utility index. Value Health.

[11] Bansback N.J., Brennan A., Ghatnekar O. (2005). Cost effectiveness of adalimumab in the treatment of patients with moderate to severe rheumatoid arthritis. Ann Rheum Dis.

[12] Harrison M.J., Lunt M., Verstappen S.M.M. (2010). Exploring the validity of estimating EQ-5D and SF-6D utility values from the Health Assessment Questionnaire in patients with inflammatory arthritis. Health Qual Life Outcomes.

[13] Lloyd S., Bujkiewicz S., Wailoo A.J. (2010). The effectiveness of anti-TNF-alpha therapies when used sequentially in rheumatoid arthritis patients: a systematic review and meta-analysis. Rheumatology.

[14] Symmons D.P.M., Tricker K., Harrison M.J. (2006). Patients with stable long-standing rheumatoid arthritis continue to deteriorate despite intensified treatment with traditional disease modifying anti-rheumatic drugs–results of the British Rheumatoid Outcome Study Group randomized controlled clinical trial. Rheumatology.

[15] Chen Y.-F., Jobanputra P., Barton B. (2006). A systematic review of the effectiveness of adalimumab, etanercept and iniximab for the treatment of rheumatoid arthritis in adults and an economic evaluation of their cost-effectiveness. Health Technol Assess.

[16] Lu G., Ades A. (2009). Modelling between-study variance structure in mixed treatment comparisons. Biostatistics.

[17] Wei Y., Higgins P.T. (2013). Bayesian multivariate meta-analysis with multiple outcomes. Stat Med.

[18] Hurst N.P., Kind P., Ruta D. (1997). Measuring health-related quality of life in rheumatoid arthritis: validity, responsiveness and reliability of EuroQoL (EQ-5D). Br J Rheumatol.

[19] Prevoo M.L.L., Vanthof M.A., Kuper H.H. (1995). Modified disease activity scores that include twenty-eight-joint counts. Development and validation in a prospective longitudinal study of patients with rheumatoid arthritis. Arthritis Rheum.

[20] Felson D.T., Anderson J.J., Boers M. (1995). American College of Rheumatology preliminary definition of improvement in rheumatoid arthritis. Arthritis Rheum.

[21] Kirwan J.R., Reeback J.S. (1986). Stanford Health Assessment Questionnaire modified to assess disability in British patients with rheumatoid arthritis. Br J Rheumatol.

[22] Spiegelhalter D, Thomas A, Best N, Lunn D. WinBUGS User Manual. 2003. Available from: http://www.mrc-bsu.cam.ac.uk/bugs/winbugs/manual14.pdf. [Accessed March 28, 2013].

[23] Spiegelhalter D.J., Abrams K.R., Myles J.P. (2004).

[24] Smith BJ. Boa: an R package for mcmc output convergence assessment and posterior inference. J Stat Software 2007;21:1–37.

[25] Minelli C., Abrams K.R., Sutton A.J., Cooper N.J. (2004). Benefits and harms associated with hormone replacement therapy: clinical decision analysis. BMJ.

[26] Turner R.M., Spiegelhalter D.J., Smith G.C.S., Thompson S.G. (2009). Bias modelling in evidence synthesis. J Royal Stat Soc A.

[27] White I. (2011). Multivariate random-effects meta-regression: update to mvmeta. Stata J.

[28] Gasparrini A. mvmeta: multivariate meta-analysis and meta-regression. R package version 0.2.3. Available from: http://cran.r-project.org/web/packages/mvmeta/. [Accessed September 12, 2013].

[29] Bujkiewicz S., Jones H., Lai M. (2011). Development of a transparent interactive decision interrogator to facilitate the decision-making process in health care. Value Health.

[30] Cooper N., Coyle D., Abrams K. (2005). Use of evidence in decision models: an appraisal of health technology assessments in the UK since 1997. J Health Serv Res Policy.

[31] European Medicines Agency. Innovative Drug Development Approaches (EMEA/127318/2007) (2007).

[32] Mason A., Drummond M., Ramsey S. (2010). Comparison of anticancer drug coverage decisions in the United States and United Kingdom: does the evidence support rhetoric?. J Clin Oncol.

[33] Coons S.J. (2009). The FDA’s critical path initiative: a brief introduction. Clin Therapeutics.

[34] Bennett A., Peterson P., Zan A., Grumley J., Panayi G., Kirkham B. (2005). Adalimumab in clinical practice. Outcome in 70 rheumatoid arthritis patients, including comparison of patients with and without previous anti-TNF exposure. Rheumatology.

[35] Bingham C.O., Ince A., Haraoui B., Keystone E.C., Chon Y., Baumgartner S. (2009). Effectiveness and safety of etanercept in subjects with RA who have failed infliximab therapy: 16-Week, open-label, observational study. Curr Med Res Opin.

[36] Bombardieri S., McKenna F., Drosos A., Michel B., Hartz D., Oezer U., Kupper H. (2007). Effectiveness of adalimumab for rheumatoid arthritis in patients with a history of TNF-antagonist therapy in clinical practice. Rheumatology.

[37] Haraoui B., Keystone E., Thorne J., Pope J., Chen I., Asare C., Leff J. (2004). Clinical outcomes of patients with Rheumatoid Arthritis after switching from Infliximab to etanercept. J Rheumatology.

[38] Hyrich K., Lunt M., Dixon W., Watson K., Symmons D. (2008). Effects of switching between anti-TNF therapies on HAQ response in patients who do not respond to their first anti-TNF drug. Rheumatology.

[39] Iannone F.T. (2007). Etanercept maintains the clinical benefit achieved by infliximab in patients with rheumatoid arthritis who discontinued infliximab because of side effects. Annals of the Rheumatic Diseases.

[40] Navarro-Sarabia F., Ruiz-Montesinos D., Hernandez B., Navarro-Compan V., Marsal S., Barcelo M. (2009). DAS-28-based EULAR response and HAQ improvement in rheumatoid arthritis patients switching between TNF antagonists. BMC Musculoskeletal Disorders.

[41] van der Bijl A.E.B. (2008). An open-label pilot study of the effectiveness of adalimumab in patients with rheumatoid arthritis and previous infliximab treatment: Relationship to reasons for failure and anti-infliximab antibody status. Clinical Rheumatology.

[42] Buch M. (2007). Therapy of patients with rheumatoid arthritis: outcome of infliximab failures switched to etanercept. Arthritis & Rheumatism (Arthritis Care & Research).

[43] Cohen G., Courvoisier N., Cohen J., Zaltni S., Sany J., Combe B. (2005). The efficiency of switching from infliximab to etanercept and vice-versa in patients with rheumatoid arthritis. Clinical & Experimental Rheumatology.

[44] Di Poi E. (2007). Switching to etanercept in patients with rheumatoid arthritis with no response to infliximab. Clinical & Experimental Rheumatology.

[45] Finckh A. (2007). B cell depletion may be more effective than switching to an alternative anti-tumor necrosis factor agent in rheumatoid arthritis patients with inadequate response to anti-tumor necrosis factor agents. Arthritis and Rheumatism.

[46] Hjardem E., Østergaard M., Pødenphant J. (2007). Do rheumatoid arthritis patients in clinical practice benefit from switching from infliximab to a second tumor necrosis factor alpha inhibitor?. Annals of the Rheumatic Diseases.

[47] Laas K.P. (2008). Clinical impact of switching from infliximab to etanercept in patients with rheumatoid arthritis. Clinical Rheumatology.

[48] Nikas S., Voulgari P., Alamanos Y., Papadopoulos C., Venetsanopoulou A., Georgiadis A., Drosos A. (2006). Efficacy and safety of switching from infliximab to adalimumab: a comparative controlled study. Ann Rheum Dis.

[49] Wick M., Linblad S., Klareskog L., van Vollenhoven R. (2005). Adalimumab (Humira) restores clinical response in patients with secondary loss of efficacy from infliximab (Remicade) or etanercept (Enbrel): results from the STURE registry at Karolinska University Hospital. Scandinavian Journal of Rheumatology.

[50] Buch M., Seto Y., Bingham S., Bejarano V., Bryer D., White J., Emery P. (2005). C-reactive protein as a predictor of infliximab treatment outcome in patients with rheumatoid arthritis. defining subtypes of nonresponse and subsequent response to etanercept. arthritis rheumatism.

[51] Karlsson J.A., Kristensen L.E., Kapetanovic M.C., Gulfe A., Saxne T., Geborek P. (2008). Treatment response to a second or third TNF-inhibitor in RA: results from the South Swedish Arthritis Treatment Group Register. Rheumatology.

[52] van Vollenhoven R., Harju A., Bran-Newark S., Klareskog L. (2003). Treatment with infliximab when etanercept has fialed or visa versa: data from the STURE registry showing that switching tumour necrosis factor alpha blockers can make sense. Ann Rheum Dis.

