# Supplementary material for: Use of Bayesian Multivariate Meta-Analysis to Estimate the HAQ for Mapping Onto the EQ-5D Questionnaire in Rheumatoid Arthritis
Source: Value Health. 2014 Jan;17(1):109–15. doi: 10.1016/j.jval.2013.11.005 (PMC3919215; doi:10.1016/j.jval.2013.11.005)
Supplement: Supplementary file 1 — Supplementary Material [file mmc1.docx]

**Appendix: Description of external summary data and resulting prior distribution for the between-study correlation.**

A sensitivity analysis was carried out to investigate the effect of the choice of parameterisation of the between-study model of the multivariate meta-analysis of the external data on resulting posterior distributions for the between-study correlations (used as prior distributions in the main meta-analysis of Lloyd data). The analysis using a multivariate Normal approximation with the spherical decomposition of the between-study covariance matrix was carried out, results of which were compared to those obtained using the product normal formulation of the between-study model.

**A1. Summary of the external data**

The external data included 29 cohorts from 16 studies listed in Table A1. Only nine of those cohorts (five studies) reported all three outcomes and data from those nine were used to construct the prior distribution for the between-study correlations in the trivariate meta-analysis. All 29 cohorts were used only in the bivariate analysis of HAQ and ACR20.

**Table A1.** Summary of the external data

| **Study** | **ACR20**  **r/n** | **ΔDAS-28**  **mean (se)** | **ΔHAQ**  **mean (se)** |
| --- | --- | --- | --- |
| Bombardieri et al. (2007) (1) | 3731 / 5711 | -2.2 ( 0.018 ) | -0.55 ( 0.01 ) |
| Cohen et al. (2004) (2) | 12 / 18 | -2.5 ( 0.17 ) | -0.6 ( 0.15 ) |
| Rau et al. (2004) i.v. (3) | 13 / 18 | -0.82 ( 0.11 ) | -0.27 ( 0.15 ) |
| Rau et al. (2004) s.c. (3) | 12 / 18 | -0.65 ( 0.18 ) | -0.1 ( 0.15 ) |
| van de Putte et al. (2003) (4) | 115 / 284 | -2 ( 0.08 ) | -0.46 ( 0.03 ) |
| van de Putte et al. (2004) 20mg eow (5) | 38 / 106 | -1.3 ( 0.16 ) | -0.29 ( 0.06 ) |
| van de Putte et al. (2004) 20mg qw (5) | 44 / 112 | -1.6 ( 0.16 ) | -0.39 ( 0.06 ) |
| van de Putte et al. (2004) 40mg eow (5) | 52 / 113 | -1.7 ( 0.15 ) | -0.38 ( 0.06 ) |
| van de Putte et al. (2004) 40mg qw (5) | 55 / 103 | -2 ( 0.16 ) | -0.49 ( 0.05 ) |
| Keystone (2004a) (6) | 238 / 419 | ̶ | -0.6 (0.03) |
| Keystone (2004b) 50mg qw (7) | 117 / 214 | ̶ | -0.5 (0.04) |
| Keystone (2004b) 25mg biw (7) | 96 / 153 | ̶ | -0.52 (0.04) |
| Klareskog (2004) ETA (8) | 169 / 223 | ̶ | -0.6 (0.04) |
| Klareskog (2004) ETA+MTX (8) | 196 / 231 | ̶ | -1 (0.04) |
| Breedveld (2006) ADA (9) | 147 / 274 | ̶ | -0.8 (0.04) |
| Breedveld (2006) ADA+MTX (9) | 195 / 268 | ̶ | -1.1 (0.04) |
| Lipsky (2000) (10) | 36 / 86 | ̶ | -0.5 (0.08) |
| Moreland (1997) (11) | 41 / 59 | ̶ | -1.3 (0.08) |
| Quinn (2005) (12) | 8 / 10 | ̶ | -1.09 (0.21) |
| StClair (2004) (13) | 219 / 351 | ̶ | -0.83 (0.04) |
| Weinblatt (2003) 20mg (14) | 33 / 69 | ̶ | -0.54 (0.07) |
| Weinblatt (2003) 40mg (14) | 45 / 67 | ̶ | -0.62 (0.08) |
| Weinblatt (2003) 80mg (14) | 48 / 73 | ̶ | -0.59 (0.06) |
| Weisman (2003) 0.25mg/kg (15) | 5 / 9 | ̶ | -0.3 (0.21) |
| Weisman (2003) 0.5mg/kg (15) | 8 / 9 | ̶ | -0.3 (0.21) |
| Weisman (2003) 1mg/kg (15) | 4 / 9 | ̶ | -0.1 (0.21) |
| Weisman (2003) 3mg/kg (15) | 7 / 9 | ̶ | -0.2 (0.21) |
| Weisman (2003) 5mg/kg (15) | 5 / 9 | ̶ | -0.3 (0.21) |
| Westhovens (2006) (16) | 199 / 343 | ̶ | -0.39 (0.03) |

Summarising the estimates for effectiveness outcomes from these studies gave average mean change from baseline in HAQ of -0.39 (sd=0.15), change from baseline in the DAS-28 of -1.64 (0.62) and odds of response measured by ACR20 of 54%; on log scale 0.17 (0.58). Figure A1 shows scatter correlation matrix plot (for the correlations between the estimates), where large correlation between the HAQ and the DAS-28 can be observed (Pearson correlation=0.9718), while correlation between HAQ and ACR20 and the DAS-28 and ACR20 are not high: -0.3369 and 0.1394 respectively.


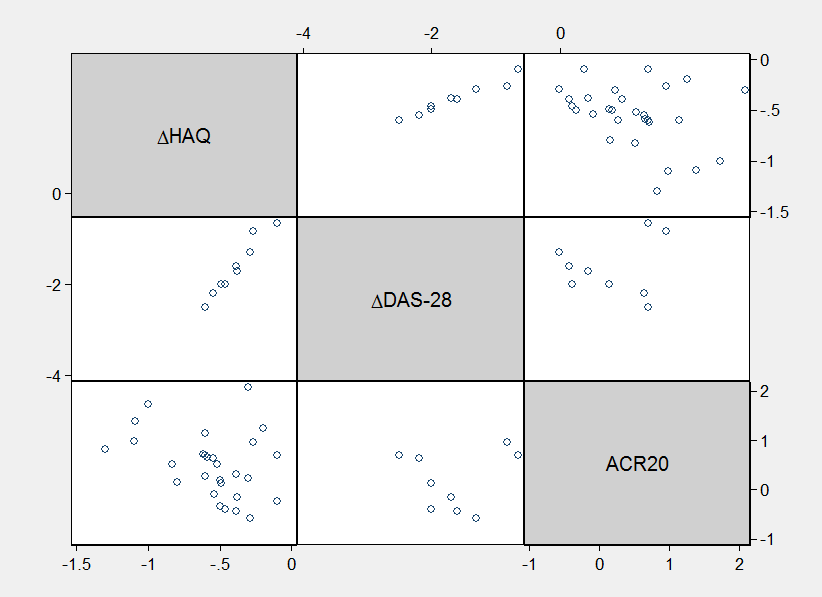


**Figure A1.** Correlation matrix for the change from baseline of ΔHAQ, ΔDAS-28 and ACR20 (on log odds scale).

**A2. Product normal parameterisation**

Using the same approach as for the analysis of Lloyd data (see main manuscript and Bujkiewicz et al (17)), the summary estimates can be modelled jointly assuming they follow trivariate normal distribution (within-study model):

$\left( \begin{matrix} \Delta H{AQ}_{i} \\ {\Delta DAS}_{i} \\ {ACR20}_{i} \end{matrix} \right)\sim N\left( \left( \begin{matrix} \mu_{Hi} \\ \mu_{Di} \\ \mu_{Ai} \end{matrix} \right),\left( \begin{matrix} \sigma_{Hi}^{2} & \sigma_{Hi}\sigma_{Di}\rho_{wi}^{HD} & \sigma_{Hi}\sigma_{Ai}\rho_{wi}^{HA} \\ \sigma_{Hi}\sigma_{Di}\rho_{wi}^{HD} & \sigma_{Di}^{2} & \sigma_{Di}\sigma_{Ai}\rho_{wi}^{DA} \\ \sigma_{Hi}\sigma_{Ai}\rho_{wi}^{HA} & \sigma_{Di}\sigma_{Ai}\rho_{wi}^{DA} & \sigma_{Ai}^{2} \end{matrix} \right) \right)$ (A1)

The underlying true effects $\mu_{Hi, Di,Ai}$ are modelled by the univariate conditional distributions (between-study model):

$\left\{ \begin{matrix} \mu_{Hi}\sim N\left( \eta_{H},\psi_{H}^{2} \right) \\ \mu_{Di}|\mu_{Hi}\sim N\left( \eta_{Di},\psi_{D}^{2} \right) \\ \eta_{Di}=\lambda_{D0}+\lambda_{D1}(\mu_{Hi}-\bar{\mu_{Hi}}) \\ \mu_{Ai}|\mu_{Hi}\sim N\left( \eta_{Ai},\psi_{A}^{2} \right) \\ \eta_{Ai}=\lambda_{A0}+\lambda_{A1}(\mu_{Hi}-\bar{\mu_{Hi}}) \end{matrix} \right.$ (A2)

and ${corr}_{DH,AH} \sim dunif\left( -1,1 \right)$, $\psi_{H,D,A}\sim N\left( 0,10 \right)I\left( 0, \right)$,$\eta_{H}\sim N\left( 0,1000 \right)$, $\lambda_{D0,A0}\sim N\left( 0,1000 \right)$,
$\lambda_{D1,A1}=\frac{\psi_{D,A}}{\psi_{H}}\frac{{corr}_{DH,AH}}{\sqrt{1-\left( {corr}_{DH,AH} \right)^{2}}}$. This parameterisation assumes a conditional independence of ΔDAS-28 and ACR20 (conditional on ΔHAQ) and hence that partial correlation (adjusted for HAQ): ${corr}_{AD\cdot H}=\frac{{corr}_{AD}-{corr}_{AH}*{corr}_{DH}}{\sqrt{1-{corr}_{AH}^{2}}\sqrt{1-{corr}_{DH}^{2}}}=0$ $CITATION DEd95 \backslash l 2057 (18)$. This implies that ${corr}_{AD}={corr}_{AH}*{corr}_{DH.}$

**A3. Spherical parameterisation**

The within-study variability is modelled by a trivariate normal distribution as in Equation (A1). The between-study variability is modelled by assuming that the true effects $\mu_{Hi, Di,Ai}$ follow common distribution:

$\left( \begin{matrix} \mu_{Hi} \\ \mu_{Di} \\ \mu_{Ai} \end{matrix} \right)\sim N\left( \left( \begin{matrix} \beta_{H} \\ \beta_{D} \\ \beta_{A} \end{matrix} \right),T \right), T=\left( \begin{matrix} \tau_{H}^{2} & \tau_{H}\tau_{D}\varrho_{HD} & \tau_{H}\tau_{A}\varrho_{HA} \\ \tau_{D}\tau_{H}\varrho_{HD} & \tau_{D}^{2} & \tau_{D}\tau_{A}\varrho_{DA} \\ \tau_{A}\tau_{H}\varrho_{HA} & \tau_{A}\tau_{D}\varrho_{DA} & \tau_{A}^{2} \end{matrix} \right)$ (A3)

The between study covariance matrix T can be decomposed using the spherical parameterisation as introduced by Lu and Ades for modelling the between-study covariance matrix in network meta-analysis (19) and adopted by Wei and Higgins for the multivariate meta-analysis (20). In this parameterisation, the covariance matrix T can be expressed as T=V^1/2^RV^1/2^, where

$V^{1/2}=\left[ \begin{matrix} \tau_{H} & 0 & 0 \\ 0 & \tau_{A} & 0 \\ 0 & 0 & \tau_{D} \end{matrix} \right]$, and the standard deviations $\tau_{H,A,D} \sim uniform \left( 0,10 \right).$

(Additional sensitivity analysis was carried out using half normal distributions on the standard deviations giving similar results.)

The correlation matrix is decomposed: R=L^T^L, where L is the upper triangle matrix in the following form:

$L=\left[ \begin{matrix} 1 & cos(\varphi_{21}) & cos(\varphi_{31}) \\ 0 & sin(\varphi_{21}) & sin(\varphi_{31})cos(\varphi_{32}) \\ 0 & 0 & sin(\varphi_{31})sin(\varphi_{32}) \end{matrix} \right]$ (A4)

and$\varphi_{ij} \in\left( 0,\pi\right)$, and therefore were assigned all $\varphi_{ij}$uniform prior distributions between 0 and π.

**A4 Comparison of results**

Table A2 shows results (mean effects and the between-study correlations) obtained from the trivariate meta-analysis model using both parameterisations: the product normal and the spherical decomposition. The estimates are presented as medians with 95% credible intervals (95% CrIs). The results from both models describe date well: they are comparable with results of summary of the data. Figures A2 and A3 show probability density distribution plots for the posterior correlations obtained from both models. The posterior distribution (used in the main analysis as the prior distribution) for the between-study correlation between the HAQ and DAS-28 obtained from the model with the spherical decomposition is more informative than the distribution obtained from the model using the product normal parameterisation. It can be justifiable to use the prior distribution obtained from the product normal formulation to allow more uncertainty in the prior distribution and to be consistent with the main analysing using the product normal parameterisation (which also enables the direct use of the external data to inform the between-study correlations in form of prior distributions).

**Table A2.** Summary of the data and results obtained from the product normal and spherical parameterisation approaches.

|  | summary of data | product normal | spherical decomposition |
| --- | --- | --- | --- |
| *summary of outcomes: mean (sd)* | | | |
| HAQ | -0.39 (0.16) | -0.4 (0.15) | -0.4 (0.2) |
| DAS-28 | -1.64 (0.62) | -1.64 (0.68) | -1.66 (0.8) |
| ACR20 | 0.17 (0.58) | 0.08 (0.68) | 0.09 (0.69) |
| *correlations* | | | |
|  | *Pearson* | *median (95% CrI)* | |
| HAQ and DAS-28 | 0.97 | 0.88 (0.14, 0.99) | 0.99 (0.71, 1.0) |
| HAQ and ACR20 | -0.34 | -0.13 (-0.8, 0.58) | -0.16 (-0.88, 0.7) |
| DAS-28 and ACR | 0.14 | *-0.09 (-0.66, 0.52)** | -0.03 (-0.9, 0.74) |

**The implied correlation between DAS-28 and ACR20 (from partial correlation equal to zero).*

**Figure A2.** Probability density plots for the correlations obtained from the model in the product normal parameterisation with implied correlation on the conditionally independent DAS-28 and ACR20.

**Figure A3.** Probability density plots for the correlations obtained from the model using the spherical parameterisation.

**A5. References**

1. Bombardieri, S., Ruiz, A. A., Fardellone, P., Geusens, P., McKenna, F., Unnebrink, K., Oezer, U., Kary, S., Kupper, H. and Burmester G. R. Effectiveness of adalimumab for rheumatoid arthritis in patients with a history of TNF-antagonist therapy in clinical practice. Rheumatology 2007, Vol. 46, pp. 1191-9.

2. Cohen, J. D., Zaltni, S., Kaiser, M. J., Bozonnat, M. C., Jorgensen, C., Daures, J. P., Sany, J. Secondary addition of methotrexate to partial responders to etanercept alone is effective in severe rheumatoid arthritis.Ann Rheum Dis 2004, Vol. 63, pp. 209-10.

3. Rau, R., Simianer, S., van Riel, P. L., van de Putte, L. B., Kruger, K., Schattenkirchner, M., Allaart, C. F., Breedveld, F. C., Kempeni, J., Beck, K. and Kupper, H. Rapid alleviation of signs and symptoms of rheumatoid arthritis with intravenous or subcutaneous administration of adalimumab in combination with methotrexate. Scand J Rheumatol 2004, Vol. 33, pp. 145–53.

4. van de Putte, L. B., Rau, R., Breedveld, F. C., Kalden, J. R., Malaise, M. G., van Riel, P. L., Schattenkirchner, M., Emery, P., Burmester, G., Zeidler, H., Moutsopoulos, H., Beck, K., and Kupper, H. Ann Rheum Dis 2003, Vol. 62, pp. 1168–77.

5. van de Putte, L. B., Atkins, C., Malaise, M., Sany, J., Russell, A. S., van Riel, P. L., Settas, L., Bijlsma, J., Todesco, S., Dougados, M., Nash, P., Emery, P., Walter, N., Kaul, M., Fischkoff, S. and Kupper, H. Efficacy and safety of adalimumab as monotherapy in patients with rheumatoid arthritis for whom previous disease modifying antirheumatic drug treatment has failed. Ann Rheum Dis 2004, Vol. 63, pp. 508–16.

6. Keystone, E. C., Kavanaugh, A. F., Sharp, J. T., Tannenbaum, H., Hua, Y., Teoh, L. S., Fischkoff, S. A. and Chartash, E. K. Radiographic, clinical, and functional outcomes of treatment with adalimumab (a human anti-tumor necrosis factor monoclonal antibody) in patients with active rheumatoid arthritis receiving concomitant methotrexate therapy: a randomized, placebo-controlled. Arthritis Rheum 2004, Vol. 50, pp. 1400-11.

7. Keystone, E. C., Schiff, M. H., Kremer, J. M., Kafka, S., Lovy, M., DeVries, T., and Burge, D. J. Once-weekly administration of 50 mg etanercept in patients with active rheumatoid arthritis: results of a multicenter, randomized, double-blind, placebocontrolled trial. Arthritis Rheum 2004, Vol. 50, pp. 353-63.

8. Klareskog, L., van der Heijde, D., de Jager, J. P., Gough, A., Kalden, J., Malaise, M., Martín Mola, E., Pavelka, K., Sany, J., Settas, L., Wajdula, J., Pedersen, R., Fatenejad, S. and Sanda, M. Therapeutic effect of the combination of etanercept and methotrexate compared with each treatment alone in patients with rheumatoid arthritis: double-blind randomised controlled trial. Lancet 2004, Vol. 363, pp. 675-81.

9. Breedveld, F. C., Weisman, M. H., Kavanaugh, A. F., Cohen, S. B., Pavelka, K., van Vollenhoven. R., Sharp, J., Perez, J. L. and Spencer-Green, G. T. The PREMIER study. A multicenter, randomized, double-blind clinical trial of combination therapy with adalimumab plus methotrexate versus methotrexate alone or adalimumab alone in patients with early, aggressive rheumatoid arthritis who had not had previo. Arthritis Rheum 2006, Vol. 54, pp. 26–37.

10. Lipsky, P. E., van der Heijde, D. M., St Clair, E. W., Furst, D. E., Breedveld, F. C., Kalden, J. R., Smolen, J. S., Weisman, M., Emery, P., Feldmann, M., Harriman, G. R. and Maini, R. N. Infliximab and methotrexate in the treatment of rheumatoid arthritis. N Engl J Med 2000, Vol. 343, pp. 1594-602.

11. Moreland, L. W., Baumgartner, S. W., Schiff, M. H., Tindall, E. A., Fleischmann, R. M., Weaver, A. L., Ettlinger, R. E., Cohen, S., Koopman, W. J., Mohler, K., Widmer, M. B. and Blosch, C. M. Treatment of rheumatoid arthritis with a recombinant human tumor. N Engl J Med 1997, Vol. 337, pp. 141–7.

12. Quinn, M. A., Conaghan, P. G., O’Connor, P. J., Karim, Z., Greenstein, A., Brown, A., Brown, C., Fraser, A., Jarret, S.and Emery, P. Very early treatment with infliximab in addition to methotrexate in early, poor-prognosis rheumatoid arthritis reduces magnetic resonance imaging evidence of synovitis and damage, with sustained benefit after infliximab withdrawal: results from a twelve-m. Arthritis Rheum 2005, Vol. 52, pp. 27-35.

13. St Clair, E. W., van der Heijde, D. M., Smolen, J. S., Maini, R. N., Bathon, J. M., Emery, P., Keystone, E., Schiff, M., Kalden, J. R., Wang, B., Dewoody, K., Weiss, R. and Baker, D. Combination of infliximab and methotrexate therapy for early rheumatoid arthritis: a randomized, controlled trial. Arthritis Rheum 2004, Vol. 50, pp. 3432–43.

14. Weinblatt, M. E., Keystone, E. C., Furst, D. E., Moreland, L. W., Weisman, M. H., Birbara, C. A., Teoh, L. A., Fischkoff, S. A. and Chartash, E. K. Adalimumab, a fully human anti-tumor necrosis factor alpha monoclonal antibody, for the treatment of rheumatoid arthritis in patients taking concomitant methotrexate: the ARMADA trial. Arthritis Rheum 2003, Vol. 48, pp. 35-45.

15. Weisman, M. H., Moreland, L. W., Furst, D. E., Weinblatt, M. E., Keystone, E. C., Paulus, H. E., Teoh, L. S., Velagapudi, R. B., Noertersheuser, P. A., Granneman, G. R., Fischkoff, S. A. and Chartash, E. K. Efficacy, pharmacokinetic, and safety assessment of adalimumab, a fully human anti-tumor necrosis factor-alpha monoclonal antibody, in adults with rheumatoid arthritis receiving concomitant methotrexate: a pilot study. Clin Ther 2003, Vol. 25, pp. 1700–21.

16. Westhovens, R., Yocum, D., Han, J., Berman, A., Strusberg, I., Geusens, P. and Rahman, M. U. The safety of infliximab, combined with background treatments, among patients with rheumatoid arthritis and various comorbidities: a large, randomized, placebo-controlled trial. Arthritis Rheum 2006, Vol. 54, pp. 1075-86.

17. B ujkiewicz S, Thompson JR, Sutton AJ, Cooper NJ, Harrison MJ, Symmons DPM, Abrams KR. Multivariate meta-analysis of mixed outcomes: a Bayesian approach. Statistics in Medicine 2013, Vol. 32, pp. 3926–3943.

18. D, Edwards. Introduction to Graphical Modelling. New York : Springer-Verlag, 1995.

19. Lu G, Ades A. Modelling between-study variance structure in mixed treatment comparisons. Biostatistics 2009, Vol. 10, pp. 792-805.

20. Wei Y, Higgins PT. Bayesian multivariate meta-analysis with multiple outcomes. Statistics in Medicine 2013, Vol. 32, pp. 2911-2934.
